# Revisiting the First Long-Term Culture of Antigen-Specific Cytotoxic T Cells

**DOI:** 10.3389/fimmu.2014.00194

**Published:** 2014-05-05

**Authors:** Kendall A. Smith

**Affiliations:** ^1^The Division of Immunology, Department of Medicine, Weill Medical College, Cornell University, New York, NY, USA

**Keywords:** cytotoxic T-lymphocytes, lymphocyte-conditioned media

Prior to the publication of this article [link to Ref. (1)], immunological dogma held that lymphocyte proliferation was only initiated and sustained by antigenic (or mitogenic) stimulation. In support of this notion, several investigators had reported that repetitive stimulation of T cells with allogeneic lymphocytes in a mixed lymphocyte culture could result in the long-term culture of alloreactive T cells for several months (2–5). With the re-addition of irradiated allogeneic stimulator cells, usually every 2 weeks, a burst of proliferation would ensue, followed by a gradual tapering of the proliferative rate of the cell population until a subsequent re-stimulation. The cellular response was assumed to be entirely antigen-driven, such that as the irradiated allogeneic stimulator cells gradually died out, the responder cells naturally defaulted to a quiescent state, since there was no longer an antigenic drive.

However, even before these reports, a decade of publications, the first in 1965, indicated that the allogeneic or mitogenic stimulation of T cells “conditioned” the media, presumably by activating the cells to release soluble mitogenic “factor activities” for T cells (6–13). At the time, the molecules responsible for the putative activities remained totally obscure, in that the available analytical and preparative biochemical methods were fairly rudimentary.

In addition to T-cell mitogenic activities, Martin Cline and David Golde had reported that human lymphocytes stimulated with phytohemagglutinin (PHA)-conditioned media that would promote the formation of granulocyte and monocyte colonies in soft agar (14). Then, Doris Morgan, a hematopoietic biologist who had been searching for a factor activity that might sustain long-term leukemia cell proliferation, reported that PHA-stimulated human lymphocyte-conditioned media promoted not leukemia cell growth, but long-term T-cell proliferation (15). It was somewhat of a surprise that mitogenic activities from lymphocyte-conditioned media could select for and support the continuous culture of human T cells for as long as 13 weeks. However, as the cellular source was bone marrow, it was assumed by many that the T cells might be immature precursors, and not mature antigen-reactive T cells.

At the time, we were in the process of examining ways to promote the generation of cytotoxic T-lymphocytes (CTL) capable of lysing murine leukemia cells. Working under the hypothesis that allogeneic leukemia cells might promote an enhanced generation of CTL lytic not only for allogeneic but also syngeneic leukemias, we had already found that secondary and tertiary allogeneic mixed tumor-lymphocyte cultures markedly augmented the lytic efficiency of CTLs, especially against syngeneic leukemia cells (16). Even so, we had not tried to culture our CTLs beyond several days, assuming that they would progressively die out. However, given Morgan’s report, we hoped that after an initial antigenic stimulation we might use lymphocyte-conditioned media to sustain long-term CTL proliferation with maintenance of lytic activity. Because concanavalin-A (Con-A) is a more potent mitogen for murine vs. human T cells, we produced Con-A T-cell supernatants, which one immunologist derisively abbreviated CATSUP, and seeded cell populations with CTL activity into 50% CATSUP. After several failed attempts by everyone in the lab, Steven Gillis, a graduate student and neophyte in cell culture, found that cells could be maintained in long-term culture provided they were seeded at low cell densities, ~5 × 10^3^ cells/mL, and never allowed to surpass ~5 × 10^5^ cells/mL (1). We speculated that perhaps low cell densities were necessary because the growth factor(s) in the CATSUP became limiting. Perhaps the high cell densities somehow consumed the activities.

The CTL lines (CTLL) retained their lytic activity upon repeated testing, and even increased their lytic efficiency up to 10-fold over several weeks of culture. Moreover, the CTLL expressed θ-antigens as expected for murine T cells, and tested negative for various histochemical stains specific for myeloid cells. Wright–Giemsa staining revealed lymphoblasts and highly vacuolated cytoplasm. Other tests at the time revealed electron-dense granules, which were subsequently found to be the cytolytic granules containing perforin and granzymes, the molecules responsible for T-cell cytolysis.

These findings were provocative for several reasons. First, the data were against the dogma that only antigens were responsible for T-cell proliferation. The data were also contrary to the notion that differentiated T-cell lytic activity and proliferation were mutually exclusive, as well as against the Hayflick hypothesis that normal cells, as opposed to malignant cells, were limited to ~50 cellular divisions before senescence ensued (17). Moreover, because the CTLL were capable of lysis of syngeneic leukemia cells, we speculated that it might be possible to generate human CTLL via allogeneic mixed tumor-lymphocyte cultures, which then could be expanded *in vitro* with CATSUP and used as adoptive immunotherapy to actually treat human leukemia.

Accordingly, we proudly submitted our manuscript to *Nature*. We felt that this paper was worthy of such a prestigious journal as *Nature* because we had shown that it is possible to culture antigen-specific functional cytolytic T cells apparently indefinitely without antigen, which was totally against the dogma that antigen was solely responsible for lymphocyte proliferation. Also, we had succeeded in selecting and culturing not only antigen-specific CTL, but also tumor antigen-specific CTLL, a holy grail of tumor immunology. However, almost by return post we received our manuscript back un-reviewed and rejected. The terse form letter from the editor said that because *Nature* received so many excellent manuscripts they could not possibly review all of them. I personally was so incensed that I immediately fired off a letter to the editor, explaining why this particular manuscript should be reviewed “by someone with more than a cursory education in immunology.” The good part of this story is that they reviewed and accepted our manuscript without changes, and it appeared on Bastille day in 1977.

This article eventually led to the generation of the first cytolytic T-cell clones, which will be the subject of another article in the series of “living immunological history” (18). However, we knew that we had difficult work ahead of us, in that our CATSUP, which was essential for our successful culture of CTLL, contained both a mitogenic lectin as well as any putative molecule(s) with soluble mitogenic activity. Which of the two was actually responsible for initiating and sustaining long-term T-cell growth had to be left to the future.

## Conflict of Interest Statement

The author declares that the research was conducted in the absence of any commercial or financial relationships that could be construed as a potential conflict of interest.
